# Prevalence of G6PD Viangchan variant in malaria endemic areas in Lao PDR: an implication for malaria elimination by 2030

**DOI:** 10.1186/s12936-019-2715-0

**Published:** 2019-03-12

**Authors:** Ken Ing Cherng Ong, Moritoshi Iwagami, Hitomi Araki, Phonepadith Khattignavong, Pheovaly Soundala, Sengdeuane Keomalaphet, Phoyphaylinh Prasayasith, Lavy Lorpachan, Phonepadith Xangsayalath, Tiengkham Pongvongsa, Bouasy Hongvanthong, Paul T. Brey, Shigeyuki Kano, Masamine Jimba

**Affiliations:** 1 Department of Community and Global Health, Graduate School of Medicine, The University of Tokyo, Tokyo, Japan; 2 SATREPS Project (JICA/AMED) for Parasitic Diseases, Vientiane, Lao People’s Democratic Republic; 3 Department of Tropical Medicine and Malaria, Research Institute, National Centre for Global Health and Medicine, 1-21-1 Toyama, Shinjuku, Tokyo 162-8655 Japan; 4 Institut Pasteur du Laos, Ministry of Health, Samsenthai Road, Ban Kao-Gnot, Sisattanak District, P.O. Box 3560, Vientiane, Lao People’s Democratic Republic; 5 National Institute of Public Health, Ministry of Health, Vientiane, Lao People’s Democratic Republic; 6 Present Address: National Center for Laboratory and Epidemiology, Ministry of Health, Vientiane, Lao People’s Democratic Republic; 7 Savannakhet Provincial Health Department, Kaysone-Phomvihan District, Savannakhet Lao People’s Democratic Republic; 8 Center of Malariology, Parasitology and Entomology, Ministry of Health, Vientiane, Lao People’s Democratic Republic

**Keywords:** Laos, Glucose-6-phosphate dehydrogenase deficiency, *Plasmodium vivax*, Malaria elimination

## Abstract

**Background:**

Primaquine is effective against the latent liver stage of *Plasmodium vivax.* Eliminating the latent liver stage of *P. vivax* is one of the necessary conditions to achieve the goal of malaria elimination in Lao People’s Democratic Republic (PDR) by 2030. However, people with glucose-6-phosphate dehydrogenase (G6PD) deficiency are at risk of haemolysis when ingesting primaquine. The aim of this study was to detect the prevalence of the G6PD Viangchan variant, which is said to be common in Lao PDR and which can result in severe haemolysis in patients exposed to primaquine.

**Methods:**

Blood samples were collected from villagers in three malaria endemic provinces: Champasak and Savannakhet in the south, and Phongsaly in the north. Each blood sample was semi-quantitatively assayed for G6PD enzyme activity using the G6PD Assay Kit-WST Lyophilized (DOJINDO Laboratories, Japan). Blood samples that were found to be G6PD deficient were sequenced to detect G6PD Viangchan mutation.

**Results:**

In total, 2043 blood samples were collected from Phongsaly (n = 426, 20.9%), Savannakhet (n = 924, 45.2%), and Champasak (n = 693, 33.9%) provinces in Lao PDR from 2016 to 2017. Of these, 964 (47.2%) were taken from male villagers and 1079 (52.8%) were taken from female villagers. G6PD Viangchan mutation was not detected in Phongsaly province in this study. In Savannakhet province, 48 of the 924 samples (45 males, 3 females) had the G6PD Viangchan mutation (n = 48, 5.2%). In Champasak province, 42 of the 693 samples (18 males, 24 females) had the G6PD Viangchan mutation (n = 42, 6.1%).

**Conclusions:**

G6PD Viangchan variant, which can cause severe haemolysis in the carrier when exposed to primaquine, was detected among 6.1% of the villagers in Champasak and 5.2% in Savannakhet but not in Phongsaly in this study. G6PD Viangchan variant might be common in the south of Laos but not so in the north. In the north, other G6PD deficiency variants might be more prevalent. However, in order not to overlook anyone and ensure a safe primaquine therapy for people living in malaria endemic areas in Lao PDR, G6PD testing is necessary.

**Electronic supplementary material:**

The online version of this article (10.1186/s12936-019-2715-0) contains supplementary material, which is available to authorized users.

## Background

Worldwide, 219 million malaria cases were reported and 435,000 lives were lost to the disease in 2017 [1]. In Lao People’s Democratic Republic (PDR), 8417 malaria cases were reported nationwide from December 2017 to November 2018 [2]. Since 2010, the proportion of *Plasmodium vivax* cases has been increasing and in 2016, 61% of the total reported malaria cases were caused by *P. vivax* [3]. Malaria is endemic throughout Lao PDR but 95% of all reported cases were from the five southern provinces of Savannakhet, Saravane, Champasak, Sekong, and Attapeu [4].

Compared to falciparum malaria, vivax malaria has received less attention due to the flawed assumption that it is less severe and less life-threatening [5]. However, recent studies have indicated that vivax malaria is not benign [6, 7]. Although *P. vivax* threatens almost 40% of the world’s population, the burden, economic impact, and severity of the disease are underestimated [6]. Unlike falciparum malaria which causes a single attack after a single infectious bite by the anopheline mosquito, vivax malaria results in multiple clinical attacks over several months up to two years after a single infectious bite [8]. Moreover, the liver reservoir of *P. vivax* in asymptomatic carriers in an endemic community may indeed be greater than expected due to its longer incubation period compared to *P. falciparum* [6, 7, 9].

Nevertheless, vivax malaria cases in Lao PDR might have been underestimated as only provincial and district hospitals are equipped with microscopy to conduct species specific analysis and treatment [10]. At the health centre level, malaria diagnosis is conducted using rapid diagnostic test (RDT) [10]. In Lao PDR, combo RDT (Malaria Ag P.f/P.v, Standard Diagnostics, Inc. Republic of Korea) is used for malaria diagnosis, which can differentiate between *P. falciparum* and *P. vivax*. However, the RDT has lower sensitivity towards detecting *P. vivax* when compared to *P. falciparum* [10, 11].

To prevent relapse caused by the dormant liver stage (hypnozoite) in vivax malaria, only one drug is available and that is primaquine [12]. In Lao PDR, a standard treatment regimen of primaquine for hypnozoite elimination is 0.25 mg/kg for 14 days. Primaquine (singlelow-dose) is also effective against the gametocyte stage of falciparum malaria and this treatment regimen is also adopted in Lao PDR [1]. However, primaquine causes acute haemolytic anaemia in patients with glucose-6-phosphate dehydrogenase (G6PD) deficiency [12]. Glucose-6-phosphate dehydrogenase is an enzyme that protects the red blood cells from oxidative stress caused by consumption of certain food or medicines [13]. The gene encoding G6PD is located on the X-chromosome and consists of 13 exons [14]. As the gene for G6PD is X-linked, the enzyme activity for males is either normal or deficient, whereas the enzyme activity for females is normal, deficient, or intermediate [15, 16]. Almost 190 G6PD mutations have been identified and most of them are missense mutations [17, 18].

Despite G6PDd being common in the Greater Mekong Subregion, information on G6PDd from the area is scant [19, 20]. However, studies have reported the presence of the Canton, Chinese-4, Kaiping, Mahidol, Union, and Viangchan variants in the Greater Mekong Subregion [21, 22]. Hence, to prevent relapse from vivax malaria, to control the spread of vivax malaria, and also to achieve malaria elimination in Lao PDR, it is necessary to prescribe primaquine to malaria patients. Therefore, to avoid any life-threatening complications, a patient’s G6PD status must first be identified before prescribing primaquine [23, 24]. This is in line with the policy adopted by the government of Lao PDR to prescribe primaquine only after the G6PD status of the patient is identified [25].

The aim of this study was to detect the prevalence of G6PD Viangchan deficiency in malaria endemic areas in Phongsaly, Savannakhet, and Champasak provinces in Lao PDR. It is hoped that the findings from this study will contribute to the decision making in the context of primaquine prescription among healthcare providers in these areas in the future.

## Methods

### Study areas and study populations

**Fig. 1:**
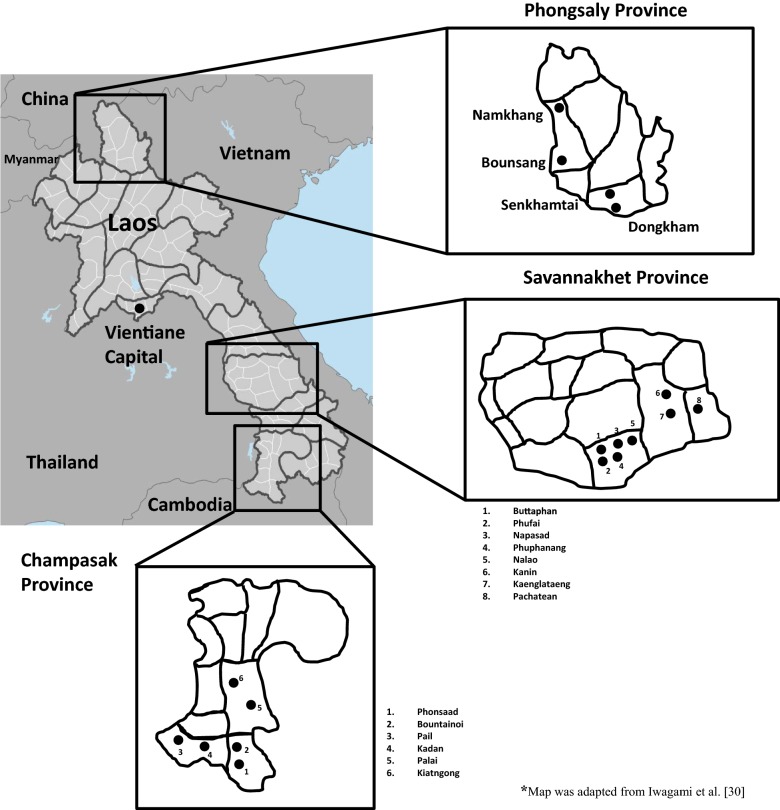
Study areas

This study was conducted in Champasak province in February 2016, Phongsaly province in February 2017, and Savannakhet province in November 2016 and October 2017. These three provinces were selected because of the high number of reported malaria cases and also a relatively high number of reported vivax malaria cases [2]. Moreover, as Lao PDR is an ethnically diverse country [26], the prevalence of G6PDd among different ethnic groups living in these three provinces was also assessed. In each province, two to three districts with the highest number of malaria cases were selected based on the data of the Center of Malariology, Parasitology and Entomology, Ministry of Health [27]. At the district level, the villages were selected after consultation with the district health office (Fig. 1).

In each village, the village head was requested to inform the entire village about the survey. As malaria affects all regardless of age and gender, all villagers who were present on the day of the survey were included.

### Blood collection procedure

Blood was collected from each villager through a finger prick. Two hundred fifty μl of blood was collected on a filter paper (FTA™ Classic Card, Whatman™, GE Healthcare Life Sciences, UK) for analysis of the G6PD gene. Ten μl of blood was used for thick and thin blood smear for malaria diagnosis by microscopy.

### G6PD enzyme activity semiquantitative test

G6PD Assay Kit-WST lyophilized (DOJINDO Laboratories, Japan) was used for enzyme activity assay. From the 10 μl of blood collected from each villager, 5 μl of blood was mixed with the substrate and dye mixture for testing and 5 μl of blood was mixed with only dye mixture for negative control according to the manufacturer’s manual. After mixing the blood with the solution, the tubes were placed in an icebox immediately to stop further reaction. The tubes were later transported to the research team’s guesthouse for further analysis within the same day of blood collection.

At the guesthouse where a makeshift laboratory had been set up, the samples were incubated for 30 min at 37.0 °C. After the incubation, the enzyme activity was measured using a NanoDrop 2000c UV–Vis Spectrophotometer (Thermo Fisher Scientific Inc. USA). Absorbance was measured at 450 nm and the background color was subtracted using the negative control. To obtain the calibration curve, a blood sample with normal G6PD activity was used as a positive control. The enzyme activities for all other samples were measured against the positive control according to the manufacturer’s instruction. Samples with enzyme activity less than 60.0% of the positive control were selected for further genetic analysis.

### Genetic analysis of the G6PD gene

The dried blood samples on the filter papers were transported from the field to the Institut Pasteur du Laos (IPL) in Vientiane for genetic analyses. DNA was extracted from the dried blood samples on the filter paper using the QIAamp DNA Mini Kit (Qiagen, Germany) according to the manufacturer’s instructions.

Exon 9 of the G6PD gene was sequenced to detect G6PD Viangchan mutation (871G > A). The primer set (Sense primer: ACCCAAGGAGCCCATTC; Antisense primer: ACACAGGGCATGCCCAGTTCTG) for Exon 9 of the G6PD gene previously reported by Matsuoka et al. was used [14]. For conventional PCR, TaKaRa Tks Gflex™ DNA Polymerase (TaKaRa Bio, Japan) was used. For DNA sequencing, BigDye^®^ Terminator v3.1 Cycle Sequencing Kit (Thermo Fisher Scientific Inc. USA) was used and subsequently the DNA sequence was read with a 3500 XL Genetic Analyzer (Applied Biosystems, Thermo Fisher Scientific Inc. USA).

The sequencing results were analysed by ClustalW in MEGA software version 7.0.21 using glucose-6-phosphate 1-dehydrogenase isoform b [*Homo sapiens*] sequence as a reference (Accession: NP_001035810.1).

### Ethical considerations

This study was approved by the National Ethics Committee for Health Research (NECHR), National Institute of Public Health (NIOPH), Ministry of Health, Lao PDR (No. 049 NIOPH/NECHR) and the Research Ethics Committee of the University of Tokyo (No. 11123). Participation was voluntary and a written informed consent was obtained from all participants and from the parents or guardians of those under 18 years of age.

**Table 1:** Summary of the villagers by sex, province, district, G6PD activity tested by DOJINDO Kit, and G6PD Viangchan mutation allelic frequencies

Province	District	Village (major ethnic group)	Total number of participated villagers by sex		G6PD Viangchan		G6PD Viangchan (%)	Hemizygous male	Heterozygous female	Homozygous female	Allelic frequency (%)	Total by province
			Male	Female	Male	Female						
Phongsaly	Bounneua	Namkhang (Akha)	34	81								
		Bounsang (Akha)	51	52								
	Khoua	Senkhamtai (Pala Akha)	55	48								
		Dongkham (Ko Akha, Tai Yang, Khmu)	39	66								426
Savannakhet	Phin	Kaenglataeng (Mangkong)	61	51	10	1	9.8	10		1	7.36	
		Kanin (Mangkong)	45	51	3		3.1	3			2.04	
	Nong	Pachatean (Mangkong)	85	95	3		1.7	3			1.09	
	Thapangthong	Buttaphan (Lao)	34	66	3		3.0	3			1.81	
		Phufai (Lao)	44	56	2		2.0	2			1.28	
		Phuphanang (Katang)	54	48	12	1	12.7	12		1	9.33	
		Nalao (Katang)	60	44	5	1	5.8	5		1	4.73	
		Napasad (Katang)	68	62	7		5.4	7			3.65	924
Champasak	Khong	Phonsaad (Lao/Pa Lao)	71	53	4	4	6.5	4	4		4.52	
		Bountainoi (Lao)	59	59	3		2.5	3			1.69	
	Mounlapamok	Pail (Khmer)	44	59	1	5	5.8	1	3	2	4.94	
		Kadan (Lao)	50	56	2	4	5.7	2	4		3.70	
	Pathoumpone	Palai (Lao)	41	62	2	4	5.8	2	2	2	4.85	
		Kiatngong (Lao)	69	70	6*	7	9.4	5	4	4	8.13	693
		Total	964	1079	63	27		62	17	11		2043

* One male villager was found to be heterozygous for G6PD Viangchan mutation so his data was included under the heterozygous female for allelic frequency calculation

## Results

In total, 2043 blood samples were collected from Phongsaly (n = 426, 20.9%), Savannakhet (n = 924, 45.2%), and Champasak (n = 693, 33.9%) provinces in Lao PDR from 2016 to 2017. Out of 2043 blood samples, 964 (47.2%) were from male villagers and 1079 (52.8%) were from female villagers. The G6PD Viangchan mutation was not detected among the 426 blood samples from Phongsaly province in this study. In Savannakhet province, 48 blood samples (45 males, 3 females) out of 924 blood samples showed the G6PD Viangchan mutation (n = 48, 5.2%). In Champasak province, 42 blood samples (18 males, 24 females) out of 693 blood samples showed the G6PD Viangchan mutation (n = 42, 6.1%) (Table 1).

The percentage of villagers carrying the G6PD Viangchan mutation ranged from 1.7% in Pachatean village, Nong district, Savannakhet province to 12.7% in Phuphanang village, Thapangthong district, Savannakhet province (Table 1).

The allelic frequencies of Viangchan mutation for each village were calculated and ranged from 1.09% in Pachatean village, Nong district, Savannakhet province to 9.33% in Phuphanang village, Thapangthong district, Savannakhet province (Table 1).

The number of malaria cases confirmed by microscopy is shown in Table 2. Information on the age and sex of the villagers is summarized in Additional file 1: Appendix S1.

**Table 2:** Number of malaria cases confirmed by microscopy

Province	District	Village (major ethnic group)	Total number of participated villagers by sex		*Pf* positive by microscopy		*Pv* positive by microscopy		Total by province
			Male	Female	Male	Female	Male	Female	
Phongsaly	Bounneua	Namkhang (Akha)	34	81					
		Bounsang (Akha)	51	52					
	Khoua	Senkhamtai (Pala Akha)	55	48			3	1	
		Dongkham (Ko Akha, Tai Yang, Khmu)	39	66					426
Savannakhet	Phin	Kaenglataeng (Mangkong)	61	51		1			
		Kanin (Mangkong)	45	51					
	Nong	Pachatean (Mangkong)	85	95	1	1			
	Thapangthong	Buttaphan (Lao)	34	66					
		Phufai (Lao)	44	56					
		Phuphanang (Katang)	54	48					
		Nalao (Katang)	60	44					
		Napasad (Katang)	68	62					924
Champasak	Khong	Phonsaad (Lao/Pa Lao)	71	53		1			
		Bountainoi (Lao)	59	59		1			
	Mounlapamok	Pail (Khmer)	44	59	1		1		
		Kadan (Lao)	50	56					
	Pathoumpone	Palai (Lao)	41	62	2	2	1		
		Kiatngong (Lao)	69	70	7	3*		1*	693
		Total	964	1079	11	9	5	2	2043

* Mixed infection, counted twice

## Discussion

The main aim of this study was to screen for the G6PD Viangchan mutation among 2043 villagers from different ethnic groups in Phongsaly, Savannakhet, and Champasak provinces. In Savannakhet province and Champasak province, 5.2% and 6.1% of the villagers were found to carry the G6PD Viangchan mutation respectively. However, G6PD Viangchan mutation was not observed among the ethnic groups in Phongsaly. This might be due to G6PD Viangchan not being common among the ethnic groups in that area. The results in this study are consistent with the predicted G6PD deficiency prevalence of 1–23% across Lao PDR [19]. As G6PD Viangchan is more common among the Lao and Thai population, this might explain why G6PD Viangchan was not detected among the population in Phongsaly province who are of a different ethnic group [28]. Severe haemolysis could occur in people with G6PD Viangchan mutation when under oxidative stress caused by agents such as primaquine [29, 30].

In this study, all the villages were in remote locations and far away from the district and provincial hospitals. In Lao PDR, G6PD testing is required before primaquine prescription since 2010 [3]. However, at the moment, G6PD rapid diagnostic tests are only available at the provincial or district hospital levels. As many patients live in remote villages, many of them are reluctant to go to either the district or provincial hospitals when advised to (personal communication with a local healthcare worker). Unless primaquine and G6PD testing are made available at the village level, elimination will not be achieved. Moreover, strict pharmacovigilance, which includes monitoring and measuring the haemoglobin level and haematocrit values, is also recommended alongside primaquine use. However, health centres at the village level still lack the capacity to conduct these tests. This also highlights the necessity of improving the quality of health centres at the village level.

Asymptomatic *Plasmodium* carriers are quite common in Lao PDR and a sizeable proportion of them are due to *P. vivax* [31]. This suggests the necessity of employing primaquine to eliminate the latent liver reservoirs to achieve the elimination goal by 2030. However, as G6PD deficiency is prevalent in malaria endemic areas in Lao PDR, and severe complications can result in the case of G6PD Viangchan mutation, G6PD screening has the potential to avoid unnecessary loss of lives in these endemic areas. In addition, as G6PD deficiency also affects the presentation of other diseases such as typhoid fever and dengue fever which are prevalent in Lao PDR, the benefits of knowing whether or not a person has G6PD deficiency could extend beyond the context of malaria [32].

In this study, the prevalence of only G6PD Viangchan was reported. However, other G6PD deficiency variants such as the Canton, Chinese-4, Kaiping, Mahidol, and Union have been reported in Lao PDR as well as other Greater Mekong Subregion countries [21, 22]. This emphasizes the importance of conducting thorough G6PD screening before primaquine prescription to ensure patient safety. Moreover, participant selection in this study was based on a voluntary basis therefore the prevalence in this study might not reflect the prevalence in the general population.

The villagers were not asked about their illness history in this study. However, recent haemolytic events can also affect a villager’s G6PD activity. A patient with G6PD enzyme deficiency who had a recent haemolytic event might have a higher proportion of young red blood cells with higher G6PD activity and this could potentially mislead result using the semiquantitative G6PD Assay Kit-WST lyophilized (DOJINDO Laboratories, Japan) [33]. This could result in the G6PD deficiency in this study being underestimated.

## Conclusions

In conclusion, G6PD Viangchan, which could cause severe haemolysis in the carrier if exposed to primaquine, was prevalent in Savannakhet and Champasak provinces, but not in Phongsaly province in this study. As Lao PDR is aiming to eliminate malaria by 2030, primaquine is indispensable. However, dispensing of primaquine without reliable G6PD testing could cause severe complications among people who have G6PD deficiency [25]. Even though blood transfusion could save lives, the procedure itself carries risks, is costly, and blood supply might be unavailable in this setting [34]. Due to the high prevalence of G6PD deficiency among people living in endemic areas, thorough and systematic G6PD screening is indispensable as the benefits could go beyond the context of malaria elimination. As strict pharmacovigilance is also recommended alongside primaquine, improving the quality of the facilities and the skills of the healthcare providers at the village level is necessary.

## Additional file

**Additional file 1: Appendix S1.** Villagers’ information.

## Abbreviations

Lao PDR: Lao People’s Democratic Republic
G6PDd: Glucose-6-phosphate dehydrogenase deficiency
